# Expression of Opsins of the Box Jellyfish *Tripedalia cystophora* Reveals the First Photopigment in Cnidarian Ocelli and Supports the Presence of Photoisomerases

**DOI:** 10.3389/fnana.2022.916510

**Published:** 2022-08-05

**Authors:** Anders Garm, Jens-Erik Svaerke, Daniela Pontieri, Todd H. Oakley

**Affiliations:** ^1^Marine Biological Section, University of Copenhagen, Copenhagen, Denmark; ^2^Department of Biology, University of California, Santa Barbara, Santa Barbara, CA, United States

**Keywords:** photopigment, box jellyfish, cubozoa, cnidaria, phototransduction, opsin phylogeny, vision

## Abstract

Cubomedusae, or box jellyfish, have a complex visual system comprising 24 eyes of four types. Like other cnidarians, their photoreceptor cells are ciliary in morphology, and a range of different techniques together show that at least two of the eye types—the image-forming upper and lower lens eyes—express opsin as the photopigment. The photoreceptors of these two eye types express the same opsin (*Tc LEO*), which belongs to the cnidarian-specific clade cnidops. Interestingly, molecular work has found a high number of opsin genes in box jellyfish, especially in the Caribbean species *Tripedalia cystophora*, most of which are of unknown function. In the current study, we raised antibodies against three out of five opsins identified from transcriptomic data from *T. cystophora* and used them to map the expression patterns. These expression patterns suggest one opsin as the photopigment in the slit eyes and another as a putative photoisomerase found in photoreceptors of all four eyes types. The last antibody stained nerve-like cells in the tentacles, in connection with nematocytes, and the radial nerve, in connection with the gonads. This is the first time photopigment expression has been localized to the outer segments of the photoreceptors in a cnidarian ocellus (simple eye). The potential presence of a photoisomerase could be another interesting convergence between box jellyfish and vertebrate photoreceptors, but it awaits final experimental proof.

## Introduction

Photosensitive organs, eyes and ocelli, have arisen many times in animal evolution (Salvini-Plawen and Mayr, 1977; Nilsson and Pelger, 1994), and recent analyses suggest that this has even happened at least nine times within the cnidarian phylum alone (Picciani et al., 2018; Miranda and Collins, 2019). In almost all known cases, opsins are used as the photopigment initially harvesting photons as a complex including a chromophore, which is most often retinal (Land and Nilsson, 2012). Although opsins diversified dramatically since their origin, many animal eyes employ opsins from two major clades, rhabdomeric opsins, which interact with G-alpha-q, and ciliary opsins, which interact with G-alpha-t, with their expression somewhat following the morphology of the photoreceptors as their names imply (Shichida and Matsuyama, 2009) [but see (Vanfleteren and Coomans, 1976; Salvini-Plawen and Mayr, 1977) for alternative viewpoints]. Recent studies proved that another clade of opsins, xenopsins, is also used in some cases as photopigments (Vöcking et al., 2017; Döring et al., 2020). Still, other opsins, including peropsins and RGR opsins, are not directly involved in light absorption but work as photoisomerases in vertebrate ciliary photoreceptors to reactivate the opsin–retinal complex (Shichida and Matsuyama, 2009). In rhabdomeric photoreceptors, light absorption does not lead to full dissociation of retinal but to a metarhodopsin complex, which is reactivated through absorption of a second photon.

In cnidarians, few experimental data are available about the details of phototransduction, but molecular studies found some of the components to indicate G-alpha-s phototransduction (Koyanagi et al., 2008; Plachetzki et al., 2010; Liegertová et al., 2015). All examined cnidarian classes express opsins, and especially in hydrozoans and anthozoans, they can be present in high numbers (Picciani et al., 2018; Gornik et al., 2021). *Cladonema radiatum* has 18 opsin genes, and *Hydra vulgaris* has as many as 42 genes (Suga et al., 2008; Macias-Munoz et al., 2019). Based on tissue or whole-body mRNA sequencing, these opsins are expressed in all body parts, but experimental evidence for their functional significance beyond gene expression is lacking in almost all cases. The known cnidarian opsins fall into three distinct clades called cnidops, anthozoan opsins I, and anthozoan opsins II, respectively, but all medusozoan opsins are in the cnidops clade (Vöcking et al., 2017; Gornik et al., 2021). Interestingly, the three clades do not seem to be closely related to each other, indicating an ancient divergence before the origin of Bilateria (Ramirez et al., 2016; Vöcking et al., 2017).

Within cnidarians - cubomedusae (or box jellyfish) - so far represent the only case of true vision with image-forming eyes that probably evolved independently of all other image-forming eyes (Nilsson et al., 2005; Garm et al., 2011; Picciani et al., 2018). These image-forming eyes, the upper and lower lens eyes, share many structural similarities with vertebrate camera-type eyes such as a hemisphere-shaped retina made of ciliary photoreceptors, a single spherical lens at least partly focusing the light onto the retina, and an adjustable pupil in the lower lens eye (Nilsson et al., 2005). Based on electrophysiological work, immunocytochemistry, microspectrophotometry, and *in situ* hybridization, the two lens eyes use opsins as the photopigment (Garm et al., 2007; Ekström et al., 2008; O'Connor et al., 2010; Bielecki et al., 2014; Liegertová et al., 2015). Even though the photoreceptors of all examined cubozoan photoreceptors are ciliary in structure, they do not express ciliary opsins. Instead, all the recovered opsins belong to the before-mentioned clade cnidops, which is so far only found in cnidarians and they express a G-alpha-s phototransduction cascade (Koyanagi et al., 2008; Picciani et al., 2018).

In addition to the two lens eyes, all known species of cubomedusae have two other eye types, the paired pit and the slit eyes, with unknown photopigments (Garm et al., 2008; Garm and Ekström, 2010). The pit eyes are structurally similar to simple ocelli found in many scyphomedusae and hydromedusae (Yamasu and Yoshida, 1973; Weber, 1981; Singla and Weber, 1982) and are putatively non-image forming, directional light meters. The slit eyes are surprisingly complex and contain four different cell types: vitreous cells, non-sensory pigment cells, pigmented photoreceptors, and non-pigmented photoreceptors. The outer segments of the photoreceptors are arranged in an asymmetric pigment groove, which might result in spatial resolution but in the vertical plane only (Garm et al., 2008). Neither pit nor slit eyes have any functional data including no data on the photopigments. The only evidence for them being photosensory comes from morphology.

In this study, we raised antibodies against the three unidentified opsins recently discovered in transcriptomic data from the Caribbean cubozoan, *Tripedalia cystophora* (Nielsen et al., 2019). We used these antibodies to examine expression not only in polyps and medusae of *T. cystophora* but also in the hydromedusa, in *Sarsia tubulosa*, and in the ocellus-carrying rhopalia of the scyphomedusae *Aurelia aurita* and *Cassiopea xamachana*. The antibodies raised against *T. cystophora* opsins only stained the medusae of *T. cystophora* and not the polyps or the tested scyphomedusae and hydromedusae. The expression patterns in *T. cystophora* allowed us to suggest specific functions for each of them.

## Materials and Methods

### Animal Cultures

Polyps and juvenile (bell diameter (BD): 1.5–2.5 mm), sub-adult (BD: 3–5 mm), and adult (BD: 6–8 mm) medusae of *T. cystophora*, Conant 1897, were obtained from cultures at the Marine Biological Section, University of Copenhagen. They were cultured in 250 l tanks with recycled seawater on a 10:14-h light–dark cycle, a temperature of 28–29 °C, and a salinity of 36–37 PSU. Medusae of *Sarsia tubulosa*, Lesson 1843, *Aurelia aurita*, L. 1758, and *Cassiopea xamachana*, Péron and Lesueur 1809, were likewise obtained from cultures at the University of Copenhagen. They were cultured in 150 l tanks with recycled sea water on a 10:14-h light–dark cycle. The temperature and salinity were 5–6°C and 24–26 PSU for *S. tubulosa*, 10–11°C and 24–26 PSU for *A. aurita*, and 24–25°C and 33–34 PSU for *C. xamachana*. All cultures were fed Selco-enriched *Artemia salina* nauplii daily.

### Custom-Made Antibodies Against Opsins

The opsins investigated in this study were originally identified from transcriptomics of juvenile and adult medusae of *T. cystophora* (Nielsen et al., 2019). Specific antibodies against three of the recovered opsins were produced in rabbits (Genosphere, Paris, France) using the following three amino acid sequences from their c-terminus:

*Opsin 1*: RPEQTSVSAPTTQAVTAANA

*Opsin 2*: ASGVQPEKENTNTVETTREP

*Opsin 3*: GLDESEIMPTEGQEPDGQPEIT.

### Immunostaining Procedure

**Figure 1:**
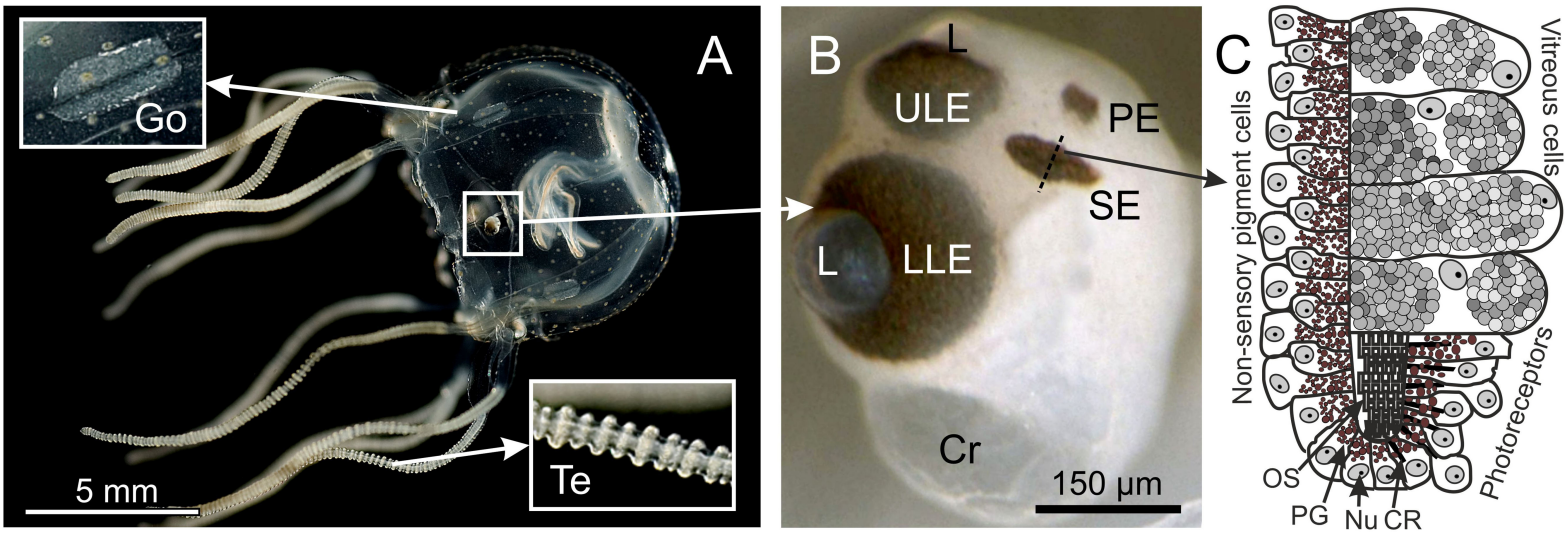
Box jellyfish *T. cystophora*. **(A)** Adult medusa of *T. cystophora* high lighting the paired gonads (Go) and tentacles (Te). **(B)** Close-up of a rhopalium showing the four eye types: upper and lower lens eye (ULE, LLE), slit eyes (SE), and pit eyes (PE). Cr, crystal; L, lens. **(C)** Schematic drawing of a cross section midways in the slit eye [broken line in **(B)**]. Note the asymmetric groove formed by the pigmented cells housing the outer segments of the ciliary photoreceptors. CR, ciliary rootlet; Nu, nucleus; OS, outer segments; PG, pigment granules. **(A)** Modified from Bielecki et al. (2013), **(C)** is modified from Garm et al. (2008).

Each of the three antibodies was used for immune staining in three polyps, 12 juvenile, four sub-adult, and two adult-male medusae of *T. cystophora* (Figure 1A). The adult medusae were dissected into quarters prior to the staining, and their rhopalia were cut off and stained separately (Figure 1B). Each quarter contained a pedalium with tentacles and a pair of gonads. Medusae were starved for 24 h prior to fixation/staining to prevent interference from their gut contents. The polyps were likewise starved but for 3 days to maximize their tentacle extension. To prevent the polyps from retracting their tentacles prior to fixation, they were anesthetized in 3.5% MgCl_2_ in seawater for 60 min prior to fixation. The polyps were kept in a dish containing ~20 ml seawater, and then, 20 ml of 7% MgCl_2_ was slowly (over 20 min) titrated into the dish. The three opsin antibodies were additionally tested on three juvenile medusae of *S. tubulosa* (BD: 2 mm), three rhopalial niches of sub-adult *A. aurita* (BD: 3–5 cm), and three rhopalial niches of sub-adult *C. xamachana* (BD: 2–4 cm).

All the materials were initially fixed in 0.1 M phosphate-buffered saline (PBS) with 4% paraformaldehyde and 5% sucrose for an hour on a rocking table at room temperature. Afterward, the tissue samples were washed 4 x 2 times with PBS buffer with triton X and bovine serum albumin (0.1 M PBS with 0.1% Triton X and 0.5% BSA) after 0, 5, 15, and 60 min.

After the last wash, the primary antibodies were added at a concentration of 1:1,000 diluted in 0.1 M PBS with 0.1% Triton X and 0.5% BSA. The incubation with the primary antibodies lasted for 72 h at 5°C in darkness followed by a second series of washes with the same time schedule as the first series. Next, the secondary antibody was added at a concentration of 1:1,000 diluted in 0.1 M PBS with 0.1% Triton X and 0.5% BSA. This incubation lasted 24 h at 5°C in darkness. After 24 h, the material was washed with pure 0.1 M PBS following the schedule above and mounted on glass slides. The material was mounted in glycol using coverslips with wax “feet,” sealed with a nail polish, and stored at 5°C in darkness until scanned (always within 5 days after preparation).

### Control Preparations

To verify that the obtained staining with the three custom-made antibodies was specific, negative controls and pre-absorption preparations were made. The pre-absorption experiments followed the general staining protocol described above with the exception that the primary antibody was mixed with the corresponding peptide for 60 min before it was included in the protocol. The peptide was diluted 1:500 by weight in 0.1 M PBS. Three juvenile *T. cystophora* medusae were used for each of the three antibody pre-absorption tests.

The negative control experiments also followed the general staining protocol with the exception that the primary antibodies were excluded from the protocol. The materials used for the negative control experiments were three juvenile *T. cystophora* medusae, three juvenile medusae of *S. tubulosa*, three rhopalial niches from *A. aurita*, and two rhopalial niches from *C. xamachana*.

### Confocal Laser Scanning Microscopy

The preparations were all scanned on a Leica SP2 microscope using 10X, 40X, or 63X oil immersion objectives. The scan depth varied between 5 and 70 μm with a z-resolution between 0.2 and 1 μm. Both single slides and maximum projections from the scans are used in the illustrations, which were prepared in Corel Graphics Suite 2020. A standard argon laser was used for both fluorescent and transmission scans.

### Opsin Phylogeny

We began with the opsin sequences and phylogeny of Picciani et al. (2018) and extracted the entire cnidops clade of opsins plus xenopsins as outgroups from the supplemental file entitled all_0512. We next searched for opsin-like genes in the recently published full genome sequence of the cubozoan *Morbakka virulenta* (Khalturin et al., 2019), using BLAST to retain the *Morbakka* sequences most similar to *T. cystophora* opsins, *Tc LEO, Tc NEO*, and *Tc GEO*, to represent each of three major groups of known box jelly opsins. After an initial alignment and phylogenetic analysis of all genes, we removed non-opsin GPCR genes of *Morbakka* based on their phylogenetic position and subsequent BLAST searches on all datasets of GenBank. This resulted in retaining two genes from *Morbakka* that fall within the cnidops clade. We then aligned all the cnidops sequences and the xenopsin outgroup sequences using MAFFT and estimated a cnidops phylogeny using IQ-TREE to select the best-fit model.

## Results

### Opsin 1—*Tc SEO*

**Figure 2:**
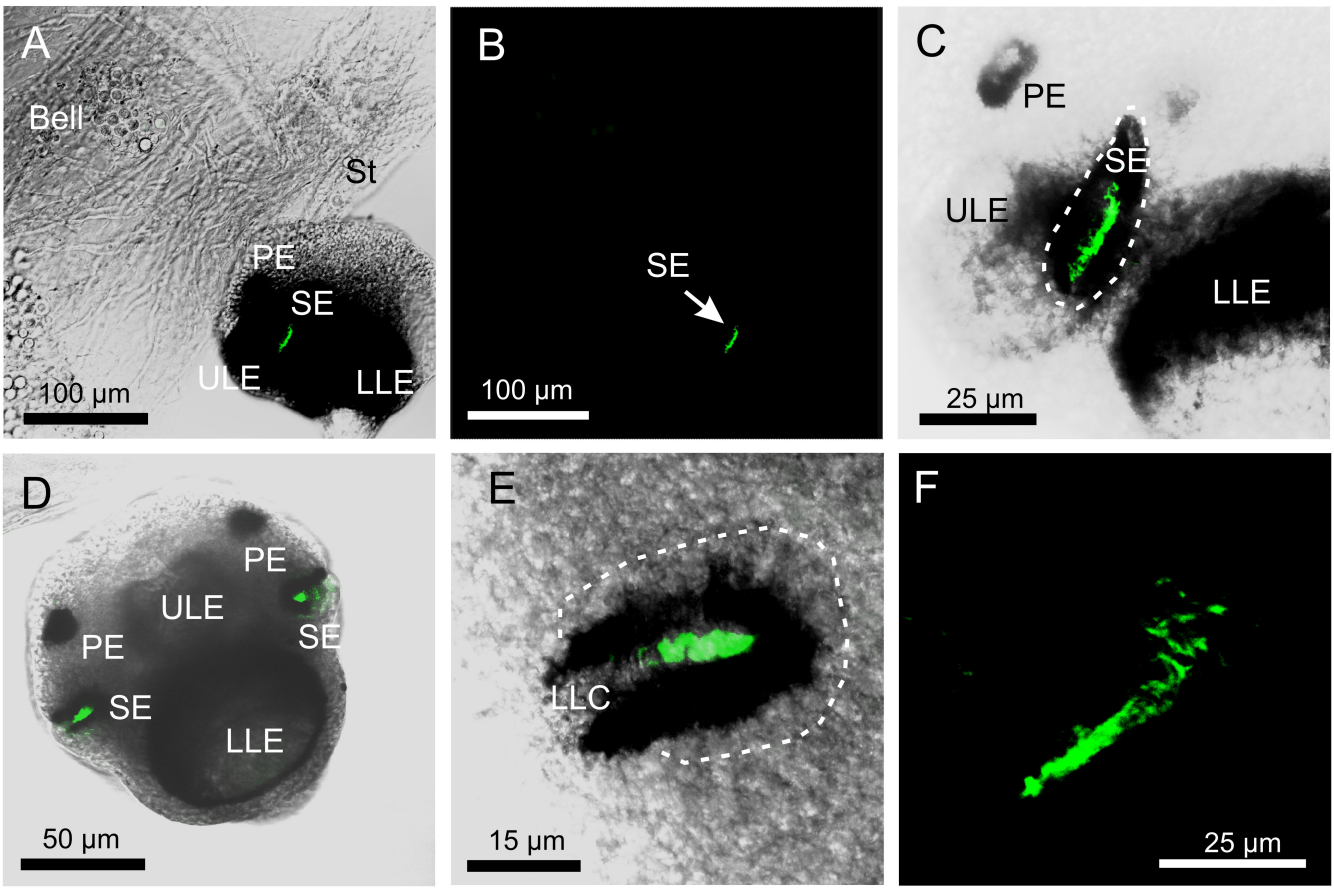
Expression of opsin 1 in *T. cystophora*. **(A)** Overlay of stain with antibody against opsin 1 (green) and transmission showing a rhopalium and parts of bell rim of a juvenile medusa. The retina of the slit eye (SE) is brightly stained. **(B)** The area in (A) only showing the immune stain. Note the complete absence of stain outside the retina of SE. **(C)** Close-up of SE from (A). The entire retina is rather evenly stained. **(D)** A juvenile rhopalium seen frontally showing identical staining in the two-slit eyes. **(E)** Close-up of left SE in (D). Note that the stain is restricted to the photoreceptors outer segments and nor the cell bodies (encircles by broken white line) or the lens-like cells (LLC) are stained. **(F)** Staining of the retina in an adult SE. LLE, lower lens eye; PE, pit eye; ULE, upper lens eye.

The antibody against opsin 1 gave a clear result in *T. cystophora*. There was no stain in the polyps, but all stages of medusae displayed a bright stain in both slit eyes (SE) and no other places (Figure 2). Accordingly, we followed our previous nomenclature (Bielecki et al., 2014) and named it *Tripedalia cystophora*slit eye opsin—*Tc SEO*. The stain was located inside the pigment screen in the area matching the outer segments of the photoreceptors (Figures 1, 2C–E). The retina was evenly stained, indicating that the antibodies had attached to all outer segments (Figure 2C).

### Opsin 2—*Tc GEO*

**Figure 3:**
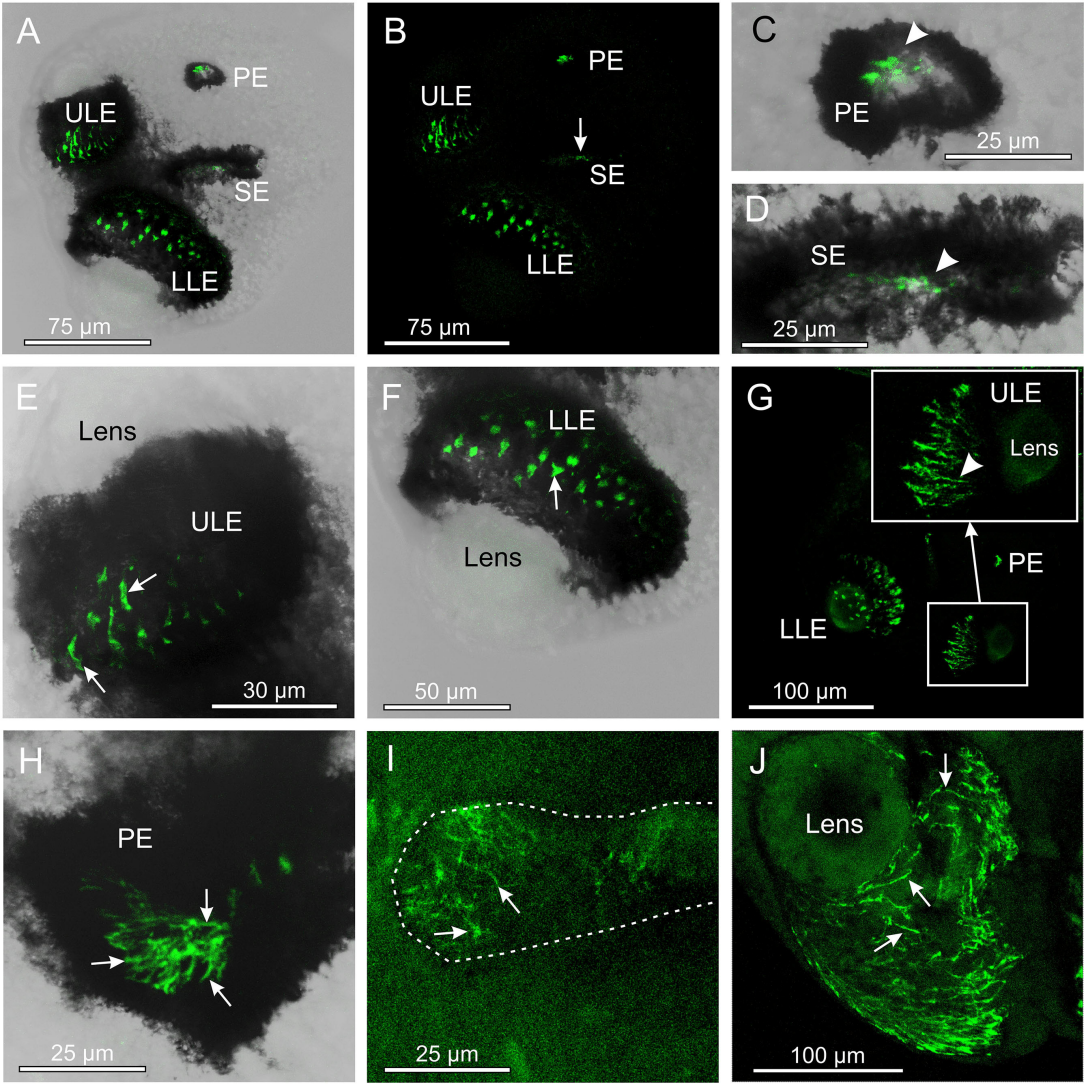
Expression of opsin 2 in *T. cystophora*. **(A)** Overlay between transmission and antibody stain of a juvenile rhopalium. Note that the retina of all eyes is stained. **(B)** The area in (A) only showing the immune stain. The slit eyes (SE) are only weakly stained (arrow). **(C)** Close-up of pit eye (PE) from (A). The stain is only seen inside the pigment screen (arrowhead) which is the outer segments of the photoreceptors. **(D)** Close-up of SE from (A). The stain is again only seen in the outer segments (arrowhead) of the photoreceptors inside the pigment screen. **(E)** Close-up of the upper lens eye (ULE) from (A). Note that the stain in the outer segments is not homogeneous but rather appears as individual lines. **(F)** Close-up of the lower lens eye (LLE) from (A). **(G)** Immuno-stain of a juvenile rhopalium clearly showing the staining pattern as lines following the orientation of the segments in the ULE (insert, arrowhead). **(H)** Stain in an adult PE also showing the expression of opsin 2 as individual lines (arrows). **(I)** The immuno-stain is also weak in the adult SE (outlines by broken white line) and appears as randomly oriented lines (arrows). **(J)** The immune stain in the adult ULE is similar to the juvenile ULE [compare with **(G)**] except for the number of stained lines (arrows) being higher.

The antibody against opsin 2 also gave a clear result in *T. cystophora*. Similar to the antibody against opsin 1, it stained the outer segments of the photoreceptors, but in all four eye types (Figure 3). Again, following our previous nomenclature, we named it Tripedalia cystophora general eye opsin—*Tc GEO*. The stain against opsin 2 was not as strong as for opsin 1, and the staining of slit eyes was especially weak (Figures 3B,D,I). Interestingly, the staining pattern in the outer segments differed when compared to opsin 1 since the stain is not homogeneous but appears as lines. In the upper lens eye (ULE), the lines are 10–30-μm long and 2–4-μm wide in the adults and 5–15-μm long and 2–4-μm wide in the juveniles. Most of the lines in ULE are more or less straight and oriented like the outer segments (Figure 3G). In the lower lens eye (LLE), which has the thickest screening pigment, the stain is harder to see. Most lines are seen through the lens of LLE and appear as dots (Figures 3F,G). The dots are 3–4-μm wide in both adults and juveniles. In the pit eyes (PE), the lines seem to have a random orientation crossing each other several times (Figure 3H). The single lines are hard to discriminate in juvenile PE, but in adults, they are more easily seen and are ~0.5-μm wide. In the SE, the stain is uneven with the lateral part of the retina being more stained (Figures 3D,I). The lines are also randomly oriented here and 5–10-μm long and ~0.5-μm wide in the adult SE.

### Opsin 3—*Tc GCO*

**Figure 4:**
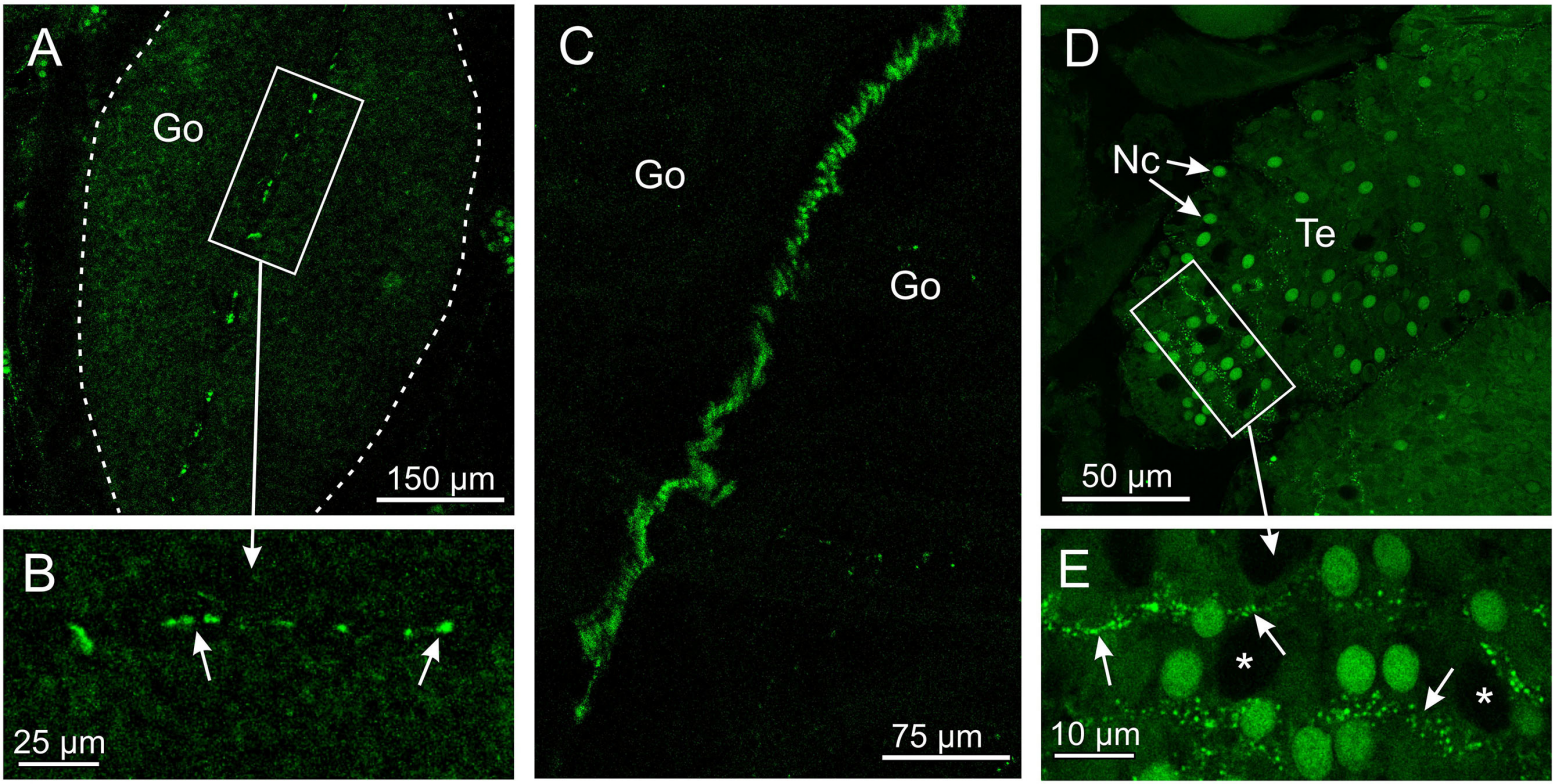
Expression of opsin 3 in *T. cystophora*. **(A)** Punctuated staining is seen along the midline of the immature gonad (Go, framed by broken white line) in the area where the interradial canal and interradial nerve are also situated. **(B)** Close-up of framed area in (A) showing the punctuated staining (arrows). **(C)** The interradial nerve stained with custom-made antibodies raised against *Tripedalia* RFamide. See Nielsen et al. (2019) for details. **(D)** Lines of punctuated staining are also found between the autofluorescent nematocyst (Nc) on the tentacles (Te). **(E)** Close-up of framed area in (C) showing lines of punctuated staining (arrows) between the Nc. Note that the larger nematocysts are not autofluorescent (asterisks).

The antibody against opsin 3 did not provide as strong a staining pattern in *T. cystophora* as the two other antibodies. The weak stain was seen in two areas and only in the medusa stage. One area is along the midline of the gonads in the area where the interradial nerve is running (Figures 4A–C). The stain was not found along the entire midline but appeared as ~20 elongated dots between 3- and 10-μm long. The other area is between the cnidocytes on the tentacles (Figures 4D,E). The stain again appeared as dotted lines but here with dots smaller than 1 μm. We named it *Tripedalia cystophora* gonad and cnidocyte opsin *Tc GCO*.

### Control Staining

**Figure 5:**
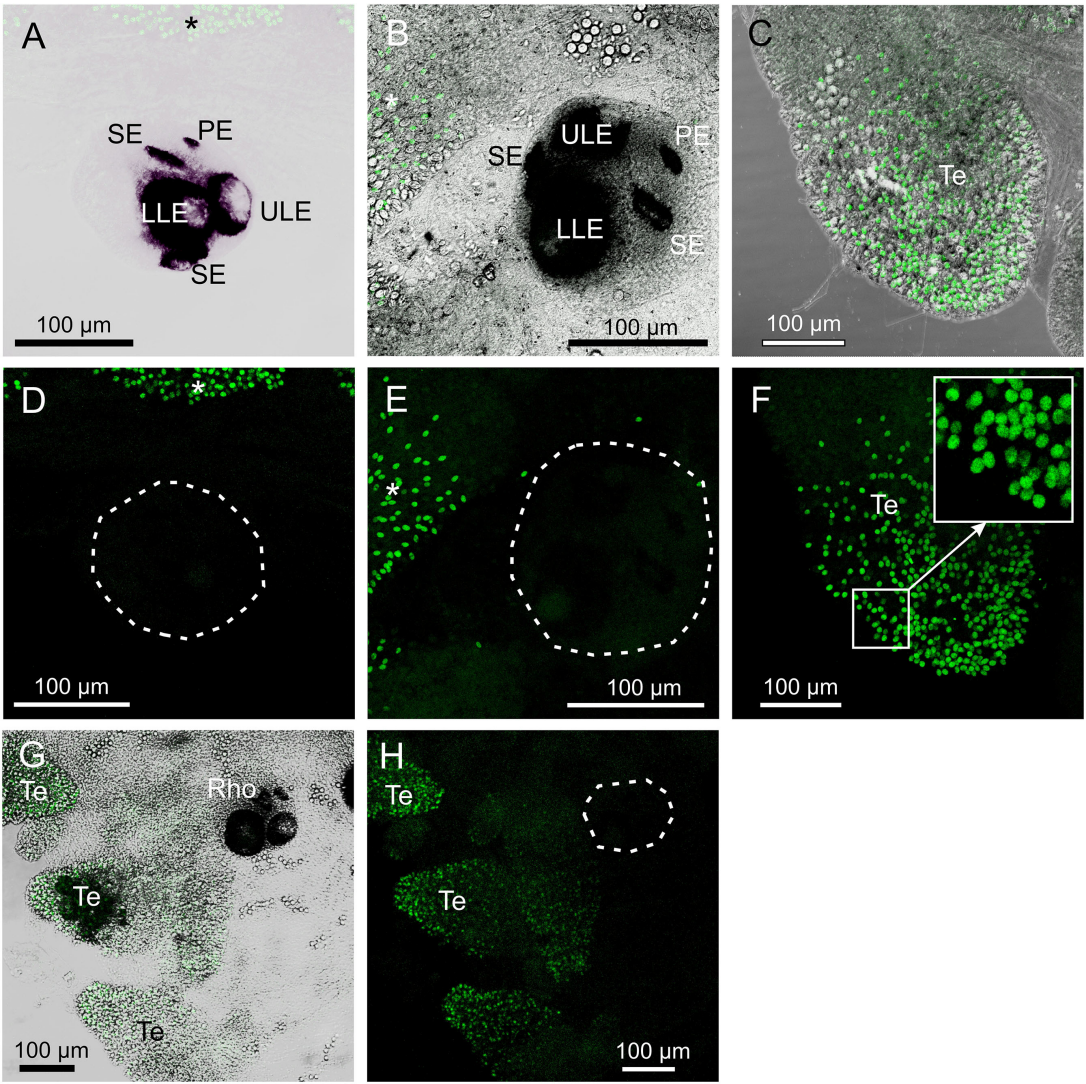
Control staining in *T. cystophora*. **(A,D)** Overlay between transmission and staining and staining alone from pre-absorption test with opsin 1 antibodies. Note the complete absence of staining in the rhopalium including the slit eye (Se). Broken white line in (D) encircles the rhopalium. Asterisks indicate autofluorescent nematocysts. **(B,E)** Overlay between transmission and staining and staining alone from pre-absorption test with opsin 2 antibodies. Note the complete absence of staining in any of the four eye types on the rhopalium [Outlined by broken white line in **(E)**]. Asterisks indicate autofluorescent nematocysts. **(C,F)** Overlay between transmission and staining and staining alone from pre-absorption test with opsin 3 antibodies. Note the absence of punctuated staining between the autofluorescent nematocyst on the tentacle (Te) (insert). **(G,H)** Negative control staining of juvenile medusa. Note the lack of staining in the rhopalium (Rho). Broken white line in **(H)** encircles the rhopalium. The only fluorescence comes from the autofluorescent nematocysts on the Te. LLE, lower lens eye; PE, pit eye; ULE, upper lens eye.

The pre-absorption tests with the antibody against opsin 1 removed all staining of the SE (Figures 5A,D). Similarly, the pre-absorption tests with the antibody against opsin 2 also removed all staining in the rhopalia (Figures 5B,E). The weak staining obtained with the antibody against opsin 3 was also absent in the tentacles (Figures 5C,F) and in the gonads (not shown) in the pre-absorption tests. Autofluorescent cnidocysts were seen in all three pre-absorption tests (Figure 5). The negative control staining without the primary antibodies displayed no stain in the rhopalia, but several autofluorescent cnidocysts were again seen (Figures 5G,H).

### Stain in *A. aurita, C. xamachana*, and *S. tubulosa*

**Figure 6:**
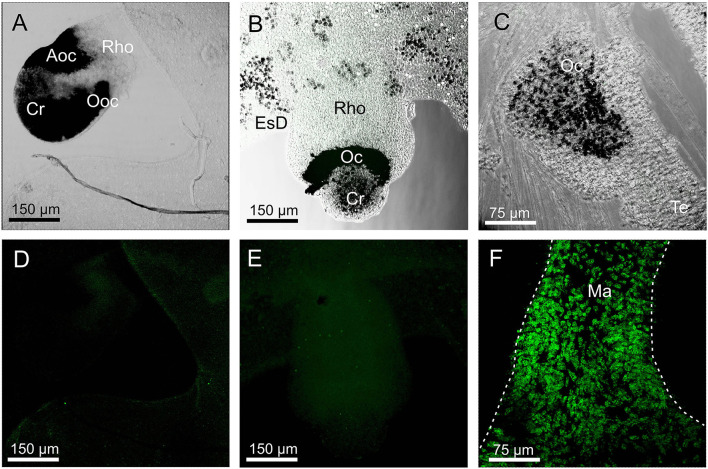
Staining with opsin 1 antibody in other medusae. **(A,D)** Overlay between transmission and staining and staining alone of a rhopalium (Rho) of *Aurelia aurita*. No staining is seen including in the aboral ocellus (Aoc) and the oral ocellus (Ooc). **(B,E)** Overlay between transmission and staining and staining alone of a rhopalium of *Cassiopea xamachana*. No staining is seen including ocellus (Oc). **(C)** Overlay between transmission and staining in the tentacular bulb of *Sarsia tubolosa*. No staining is seen including ocellus (Oc). **(F)** A huge number of autofluorescent cells are found on the middle part of the manubrium (Ma, outlined by broken white line) of *S. tubolosa*. Cr, crystal; EsD, endosymbiotic dinoflagellates; Te, tentacle.

None of the three opsin antibodies provided any specific stain in *A. aurita, C. xamachana*, and *S. tubulosa* (Figure 6). In *S. tubulosa*, epithelial cells in the middle part of the manubrium did fluoresce in all three cases, but we interpret this as autofluorescence because the same area also has fluorescence in the negative controls (not shown). Furthermore, like for *T. cystophora*, many of the cnidocysts were autofluorescent.

### Opsin Phylogeny

**Figure 7:**
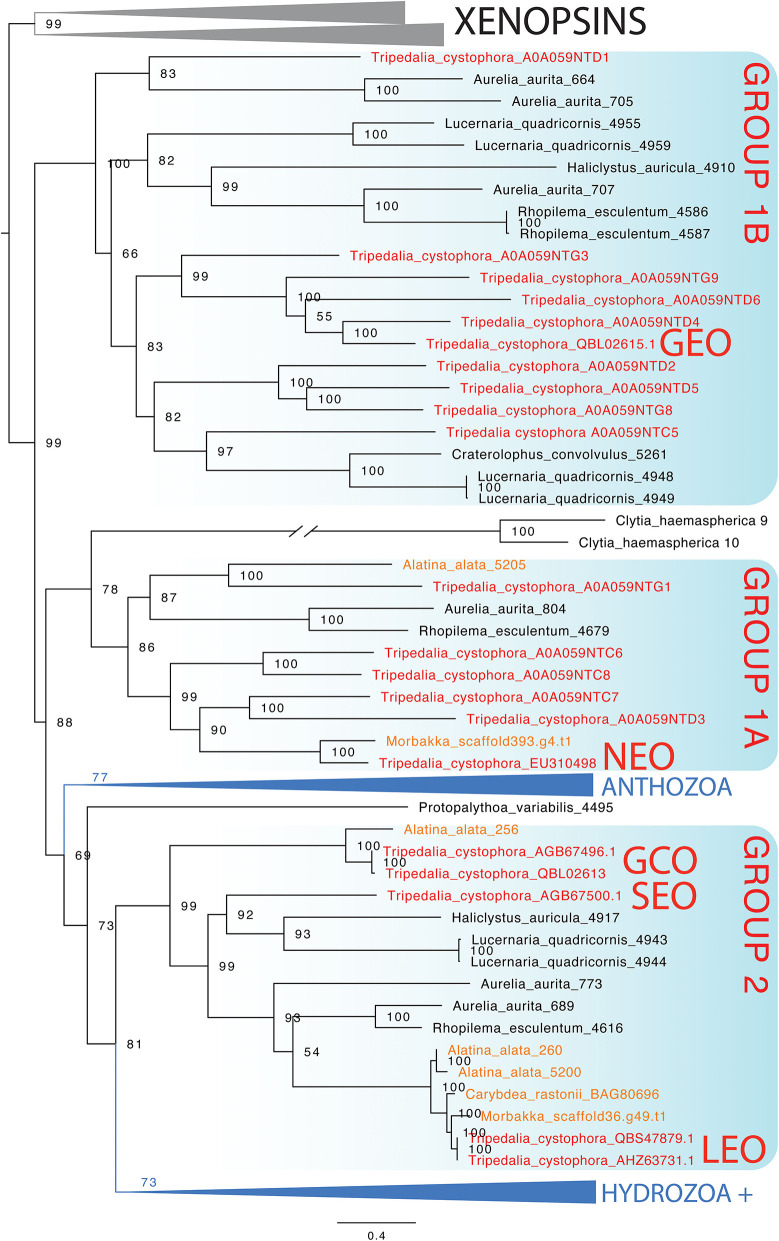
Maximum likelihood phylogenetic analysis of known opsins of the box jellyfish *Tripedalia cystophora*. We combined 21 protein sequences of opsin of *T. cystophora* (red) with other known cnidops, including those of *Alatina alata* (orange), and xenopsins (outgroup in gray), then added putative *Morbakka* opsin (orange)—for multiple alignments using MAFFT. We then selected the best-fit model in IQ-TREE for likelihood and UFBoot phylogenetic analyses. Illustrated is the bootstrap consensus with numbers at nodes showing bootstrap proportions. Two large clades are collapsed, one containing Anthozoa cnidops, and the other containing mainly Hydrozoa cnidops (see Supplementary Material for full phylogeny). Cubozoan opsins fall into three clades, somewhat consistent with previous analyses, which we label here as Group 1a, Group 1b, and Group 2 (Liegertová et al., 2015). Our antibody experiments labeled *Tripedalia cystophora* slit eye opsins—*Tc SEO* (Antibody 1), labeled *Tripedalia cystophora* general eye opsins—*Tc GEO* (Antibody 2), and labeled *Tripedalia cystophora* gonads and cnidocyte opsins—*Tc GCO* (Antibody 3). Note that both *Tc LEO* and *Tc GCO* are represented. These originate from two different studies and putatively represent two different alleles of the same opsin gene.

In the maximum likelihood phylogeny, the opsin-containing sequence used to create the opsin 1 (*Tc SEO*) antibody clusters closest to the only other ocular opsin known from *Tripedalia*, lens eye opsin (*Tc LEO*) (Figure 7). This clade, previously called “Group 2” (Liegertová et al., 2015), also contains sequences from three other cubozoan opsin genes from *Alatina alata* and *Carybdea rastoni*, three opsin genes from the scyphozoans *A. aurita* and *Rhopilema esculentum*, and two opsin genes from the stauromedusae *Haliclystus auricula* and *Lucernaria quadricornis* (Figure 7). The sequence of the opsin 3 (*Tc GCO*) gene is found within Group 2 in a sister clade to these ocular opsins and is closely related to an opsin from *A. alata* (Figure 7). Interestingly, the sequence of the opsin 2 (*Tc GEO*) gene is only distantly related to the two other opsin genes in this study and was found in a cluster together in a clade previously called Group 1b with other *Tripedalia* opsins and some opsins from scyphomedusae and stauromedusae (see also Supplementary Figures 1, 2 and Supplementary Table 1 for extended phylogenies and *Tripedalia* opsin names).

## Discussion

Opsins form the basis of vision in almost all examined eyes, but many animals contain a surprisingly high number of opsin genes, suggesting functional diversification. This is not least the case for cnidarians, and here, we used custom-made antibodies to examine the expression patterns of three opsins found in transcriptomic data from the box jellyfish *T. cystophora*. These expression patterns allowed us to suggest functions for each of them, supporting functional diversification, since they are putative visual pigment, photoisomerase, and extraocular photopigment, respectively.

### Visual Pigment of the Slit Eyes

The expression pattern of opsin 1 (*Tc SEO*) strongly suggests that this is the photopigment of slit eye photoreceptors. The staining coincides precisely with the known arrangement of the outer segments in the slit eye (Garm et al., 2008). Furthermore, it is a very homogenous stain, which is expected of the photopigments since they are densely packed in the outer segments (Nilsson, 2013). Interestingly, there is a distinct difference in the staining pattern when compared to the anti-opsin stain of putative *Tc LEO* previously obtained from the lens eyes of *T. cystophora* (Ekström et al., 2008). In the photoreceptors of the lens eyes, the opsin antibody stains both the outer segments and the cell bodies where the opsins are synthesized. This is not the case with the slit eye photoreceptors, suggesting that the c-terminus of the opsin (which the opsin 1 (*Tc SEO*) antibodies are raised against) could be hidden from the antibodies prior to opsin entering the outer segments. Two possible explanations for this could be that the c-terminus is folded differently while in the cell body or that the antibodies cannot enter the vesicles which hold the opsin during the transport to the outer segments. The uniform stain of the entire retina also strongly suggests that all photoreceptors express the same opsin and thus that the slit eyes alone do not support color vision, in contrast to an earlier suggestion based on the presence of two morphologically distinct photoreceptor types (Garm et al., 2008). Adding receptor-specific color filters could still result in color vision despite expressing a single opsin only, but there are no indications of such filters in the slit eyes (Garm et al., 2008).

To our knowledge, this is the first study to identify the photopigment of a cnidarian ocellus and thereby also demonstrate that they are opsin based like nearly all other known eyes and ocelli in the animal kingdom (Land and Nilsson, 2012). Several previous molecular studies showed that medusae with ocelli in both Hydrozoa and Scyphozoa have many opsin genes (Suga et al., 2008; Picciani et al., 2018) but their expression in the photoreceptor outer segments has not been shown before. Opsins are associated with the photoreceptors of the hydromedusa *Cladonema radiatum*, but the expression was not seen in the outer segments only in the cell body, leaving the function uncertain. In addition to molecular analyses, the photopigment of cnidarian ocelli was examined in two species of hydromedusae using electrophysiology (Weber, 1982a,b). The spectral sensitivities from those recordings do support the presence of a single opsin in both cases, even though the data were not analyzed to address this.

It is not surprising that the antibody against opsin 1 (*Tc SEO*) did not stain the photoreceptors in the ocelli of the hydromedusae, *S. turbulosa*. In the opsin phylogeny, the hydrozoan opsins cluster outside the cubozoan opsins with the interesting exception of a couple of *Clytia* opsins. This means that the *Sarsia* opsins are likely too different structurally to be recognized by specific antibodies raised against cubozoan opsins, which was confirmed when we compared the sequences of the c-termini (not shown). Our phylogeny did show that opsin 1 (*Tc SEO*) is somewhat closely related to both the *Tripedalia* lens eye opsin (*Tc LEO*) and two opsin genes from *A. aurita*, but no cross-reactions were seen in the lens eyes or the rhopalia of *A. aurita*. Despite this relationship, the c-termini of the opsins are quite different across species. Still, it is interesting that the two known cnidarian ocular opsins cluster closely together, as it could indicate that opsins in this cluster are photoreceptor photopigments. This would pinpoint the most likely candidates for ocular opsins in *A. aurita, R. esculentum*, and the putative ocelli of stauromedusae.

### Potential Photoisomerase

The staining pattern of the antibody against opsin 2 (*Tc GEO*) is highly interesting. All ocular photoreceptors in the rhopalia of *T. cystophora* seem to be stained, which can indicate one of at least three possibilities: (1) It is the photopigment of a separate type of photoreceptor present in all eyes/ocelli, (2) it is the conserved half of an opsin complex-forming dimer in all ocular photoreceptors, or (3) it is a photoisomerase reactivating the photopigments in all ocular photoreceptors. We found the third option the most likely.

If opsin 2 (*Tc GEO*) is the photopigment of a separate class of photoreceptors, and if it has a different lambda-max value, this could imply color vision in at least the slit eyes and the lens eyes. However, we do not favor this hypothesis. Nothing is known about the physiology of the photoreceptors of the slit eyes, but all available data strongly support the presence of a single photoreceptor type in the lens eyes (Garm et al., 2007; Ekström et al., 2008; O'Connor et al., 2010). Furthermore, the details of the staining patterns indicate that opsin 2 (*Tc GEO*) is situated centrally in the outer segments of the photoreceptors but not in the membrane where the photopigments are normally found.

Dimerization of opsins is a very interesting and highly debated topic, but we do not favor this hypothesis to explain the staining pattern of opsin 2 (*Tc GEO*). Opsins might form dimers similar to the GPCRs involved in insect olfaction (Rützler and Zweibel, 2005; Zhang et al., 2016). The dimerization is potentially important both for successful transportation and integration in the outer segments and for activation of the transduction cascade (Zhang et al., 2016). Still, if opsin 2 (*Tc GEO*) forms heterodimers with opsin 1 (*Tc SEO*) in the slit eyes and LEO in the lens eyes, then we would expect opsin 2 (*Tc GEO*) to be expressed in similar patterns as opsin 1 (*Tc SEO*) and LEO in the slit eye and LLE, respectively, and this is not the case (current results, Ekström et al., 2008).

We hypothesize that opsin 2 (*Tc GEO*) functions as a photoisomerase, reactivating the photopigments in the photoreceptors. Bleaching experiments in another species of box jellyfish, *C. bronzei*, hinted that reactivation does not happen through a light-activated metarhodopsin and thus likely involves a photoisomerase as in vertebrate and cephalopod photoreceptors (Shichida and Matsuyama, 2009; O'Connor et al., 2010; Vöcking et al., 2021). All photoreceptors in *T. cystophora* could use the same photoisomerase, which matches the staining of opsin 2 (*Tc GEO*) we found in all eyes/ocelli. Furthermore, a photoisomerase is likely not bound in the cell membrane but is instead found internally in the cells (Smith and Goldsmith, 1991), which also matches the present staining of opsin 2 (*Tc GEO*). Finally, function as photoisomerase instead of a photopigment is supported by opsin 2 (*Tc GEO*) being in a different evolutionary clade within cnidops, being only distantly related to the known visual opsins in *T. cystophora*.

Perhaps counter to a photoisomerase hypothesis, the extraocular photoreceptors in the rhopalium (Bielecki et al., 2014) and in the tentacles and gonads (current study) are not stained by the opsin 2 (*Tc GEO*) antibody. However, this could also be due to a low amount of opsin/photoisomerase in these cells, resulting in staining that is too weak to be convincingly detected or could indicate that the extraocular opsins are reactivated by a different mechanism.

### Extraocular Photoreceptors

The antibody against opsin 3 (*Tc GCO*) only stains structures outside the rhopalium, cells we believe to be extraocular photoreceptors. The neurite-like structures stained in the tentacles are perhaps involved in modulating cnidocyte discharge based on the light level. In a diversity of polyp stages across cnidarian taxa, the discharge of the nematocytes on the tentacles is influenced by the ambient light (Plachetzki et al., 2012; Picciani et al., 2021). We expect this function to be present in most cnidarians including box jellyfish, which display a strict diurnal rhythm (Garm et al., 2012), and the staining patterns on the tentacles in between the nematocytes are exactly how we would expect the putative opsin-carrying neurons to be arranged. The weak staining also matches an expectation of a low concentration of opsin in extraocular photoreceptors used to detect the time of day (Nilsson, 2013). Furthermore, the presence of the retinal binding residue LY296 supports the function as that of a photoreceptor (Land and Nilsson, 2012).

The stain in the area of the interradial nerve restricted to where it passes the gonad indicates that opsin 3 (*Tc GCO*) might be involved in reproduction. This is supported by juvenile and sub-adult specimens showing no stain in this area. *T. cystophora* has internal fertilization following an actual mating, so light control of gamete release is not a likely function of opsin 3 (*Tc GCO*) (Werner, 1971, 1973). In the hydromedusae, *Clytia hemisphaerica* and *Cladonema radiatum*, specific opsins are also expressed in the gonads and they are involved in gamete maturation (Suga et al., 2008; Artigas et al., 2018). In *C. hemisphaerica*, the system has been characterized in detail, and here, the opsin is expressed in the epidermal cells of the gonads along with a maturation-inducing hormone (MIH). This putatively allows for direct light control of MIH release and thus gamete maturation (Artigas et al., 2018). Little is known about gamete maturation in *T. cystophora* and about what hormones might influence this. If opsin 3 (*Tc GCO*) is involved in gamete maturation, it operates in a different way than in *C. hemispaerica*, since it is not expressed in the gonad as such but rather in the space between the two hemi-gonads in the area of the interradial nerve. Still, personal observation from their natural habitat in the Puerto Rican mangroves shows that medusa size, rather than light level or day length, seems to control when gametes/gonads start to develop, and at least male medusae can carry ripe spermatozeugmata for days (Helmark and Garm, 2019). A third option is that opsin 3 (*Tc GCO*) is not a photoreceptor but has another function, which is yet to be determined.

### Opsin Phylogeny

An open question in cnidarian biology is which opsins are expressed in eyes and ocelli of different clades. This is particularly interesting because eyes and ocelli originated multiple times convergently in medusozoa, raising the question of whether the same opsin ortholog is expressed each time. Unfortunately, expression data for cnidarian eyes are mainly missing, but our current gene phylogeny presents some candidates for future tests. Namely, Group 2 opsins contain *Tripedalia* lens eye opsins (*Tc LEO*) and slit eye opsins (*Tc SEO*), raising the possibility that this is a clade of ocular opsins. The clade also contains opsins from *Aurelia* and *Rhopilema*, which we consider to be prime candidates as ocular opsins, even though those scyphozoan eyes may be non-homologous to cubozoan eyes (Picciani et al., 2018). The Group 2 clade also contains opsins from two staurozoans. Some staurozoans have dark pigment spots consistent with their function as photoreceptive organs (Miranda and Collins, 2019). These Group 2 opsins are candidates for expression in these pigment spots.

Our phylogeny of cnidarian opsins focused on *Tripedalia* is consistent with previous studies that found a diverse assemblage of opsins in box jellyfish (Liegertová et al., 2015; Ramirez et al., 2016). Two groups of Tripedalia opsins (Group 1 and Group 2) are distantly related to each other, with Group 2 being related most closely to a large clade of hydrozoan opsins. In our analysis, Group 1 opsins are paraphyletic with Group 1B as the sister group to all the other cnidops. This is a rather surprising result because it implies that 1A and 1B were lost independently from Anthozoa and Hydrozoa. An alternative interpretation is that the gene tree is challenging to root properly. We chose xenopsins as the outgroup to cnidops based on previous studies (Ramirez et al., 2016; Vöcking et al., 2017), and so we do not believe there is a better choice of outgroup opsins. Future studies could explicitly consider gene duplication and loss events when constructing the gene tree.

## Data Availability Statement

Data and phylogenetic analyses can be found online at: https://github.com/ostratodd/Tripedalia_opsin_expression_2022.

## Author Contributions

AG conceptualized the work, designed the experiments, wrote the initial version of the manuscript, and designed the figures. J-ES and DP collected and analyzed the immunocytochemical data. TO performed the phylogenetic analysis. AG and TO financed the work. All authors contributed to the final version.

## Conflict of Interest

The authors declare that the research was conducted in the absence of any commercial or financial relationships that could be construed as a potential conflict of interest.

## Publisher's Note

All claims expressed in this article are solely those of the authors and do not necessarily represent those of their affiliated organizations, or those of the publisher, the editors and the reviewers. Any product that may be evaluated in this article, or claim that may be made by its manufacturer, is not guaranteed or endorsed by the publisher.

## Supplementary Material

Supplementary Figure 1 Maximum likelihood phylogenetic analysis to discover opsins from the genome of *Morbakka*. We searched for opsin-like genes in the recently published full genome sequence of the cubozoan *Morbakka virulenta* (Khalturin et al., 2019), using BLAST to retain the *Morbakka* sequences most similar to *Tripedalia cystophora* opsins *Tc LEO, Tc NEO*, and *Tc GEO*, which represent each of the major groups 1a, 1b, and 2. Such similarity searches usually retain non-opsin GPCR genes. To determine non-opsins, we combined the *Morbakka* genes with our cnidops and xenopsin dataset (see Figure 7 and Supplementary Figure 2) and estimated this phylogeny in IQ-TREE using the best-fit model, illustrated here rooted with a clade of 16 *Morbakka* genes (magenta). These 16 genes are non-opsin GPCRs that we removed for final analyses. The topology of the overall tree shown here is different from the final, probably because adding non-opsin genes to opsins destabilizes the opsin ingroup (Plachetzki et al., 2007). This tree also contains duplicated genes of *Tripedalia cystophora* (black) that were removed before the final analysis. Multiple sequences are very similar to other sequences and are probably allelic variants, but that cannot be determined with certainty without genome sequencing. The scale bar indicates amino acid substitutions per site.

Supplementary Figure 2 Full phylogenetic tree for our xenopsin + cnidops dataset. See Supplementary Figure 1 and main text for methodological details. The scale bar indicates amino acid substitutions per site.
