# Corrigendum: Expression of opsins of the box jellyfish *Tripedalia cystophora* reveals the first photopigment in cnidarian ocelli and supports the presence of photoisomerases

**DOI:** 10.3389/fnana.2022.1028092

**Published:** 2022-09-08

**Authors:** Anders Garm, Jens-Erik Svaerke, Daniela Pontieri, Todd H. Oakley

**Affiliations:** ^1^Marine Biological Section, University of Copenhagen, Copenhagen, Denmark; ^2^Department of Biology, University of California, Santa Barbara, Santa Barbara, CA, United States

**Keywords:** photopigment, box jellyfish, cubozoa, cnidaria, phototransduction, opsin phylogeny, vision

In the published article, there was an error in the legend for **Figure 1** as published. Credits are missing for the picture in **(A)**. The corrected legend appears below.

“Box jellyfish *T. cystophora*. **(A)** Adult medusa of *T. cystophora* high lighting the paired gonads (Go) and tentacles (Te). **(B)** Close up of a rhopalium showing the four eye types: upper and lower lens eye (ULE, LLE), slit eyes (SE), and pit eyes (PE). Cr, crystal; L, lens. **(C)** Schematic drawing of a cross section midways in the slit eye [broken line in **(B)**]. Note the asymmetric groove formed by the pigmented cells housing the outer segments of the ciliary photoreceptors. CR, ciliary rootlet; Nu, nucleus; OS, outer segments; PG, pigment granules. **(A)** Modified from Bielecki et al. (2013), **(C)** is modified from Garm et al. (2008).”

The authors apologize for this error and state that this does not change the scientific conclusions of the article in any way. The original article has been updated.

## Publisher's note

All claims expressed in this article are solely those of the authors and do not necessarily represent those of their affiliated organizations, or those of the publisher, the editors and the reviewers. Any product that may be evaluated in this article, or claim that may be made by its manufacturer, is not guaranteed or endorsed by the publisher.
