# Supplementary figures and images for: Expression of Opsins of the Box Jellyfish Tripedalia cystophora Reveals the First Photopigment in Cnidarian Ocelli and Supports the Presence of Photoisomerases
Source: Front Neuroanat. 2022 Aug 5;16:916510. doi: 10.3389/fnana.2022.916510 (PMC9389615; doi:10.3389/fnana.2022.916510)

XENOPSINS

GROUP 1B

1A

ANTHOZOA

GROUP 2

HYDROZOA +

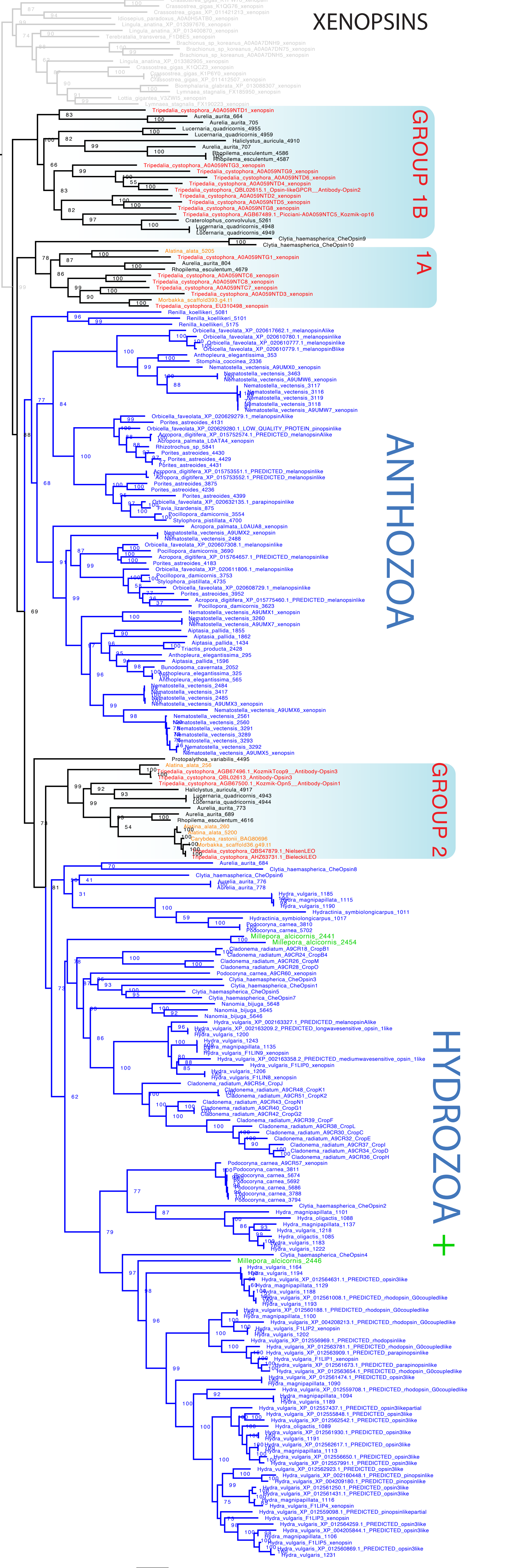

Supplement: Supplementary Figure 1 — Maximum likelihood phylogenetic analysis to discover opsins from the genome of Morbakka. We searched for opsin-like genes in the recently published full genome sequence of the cubozoan Morbakka virulenta (Khalturin et al., 2019), using BLAST to retain the Morbakka sequences most similar to Tripedalia cystophora opsins Tc LEO, Tc NEO, and Tc GEO, which represent each of the major groups 1a, 1b, and 2. Such similarity searches usually retain non-opsin GPCR genes. To determine non-opsins, we combined the Morbakka genes with our cnidops and xenopsin dataset (see Figure 7 and Supplementary Figure 2) and estimated this phylogeny in IQ-TREE using the best-fit model, illustrated here rooted with a clade of 16 Morbakka genes (magenta). These 16 genes are non-opsin GPCRs that we removed for final analyses. The topology of the overall tree shown here is different from the final, probably because adding non-opsin genes to opsins destabilizes the opsin ingroup (Plachetzki et al., 2007). This tree also contains duplicated genes of Tripedalia cystophora (black) that were removed before the final analysis. Multiple sequences are very similar to other sequences and are probably allelic variants, but that cannot be determined with certainty without genome sequencing. The scale bar indicates amino acid substitutions per site. [file Data_Sheet_1.PDF]

NON-OPSIN GPCRs

HYDROZOA +

GROUP 2

HYDROZOA +

GROUP 1B

XENOPSINS

1A

ANTHOZOA

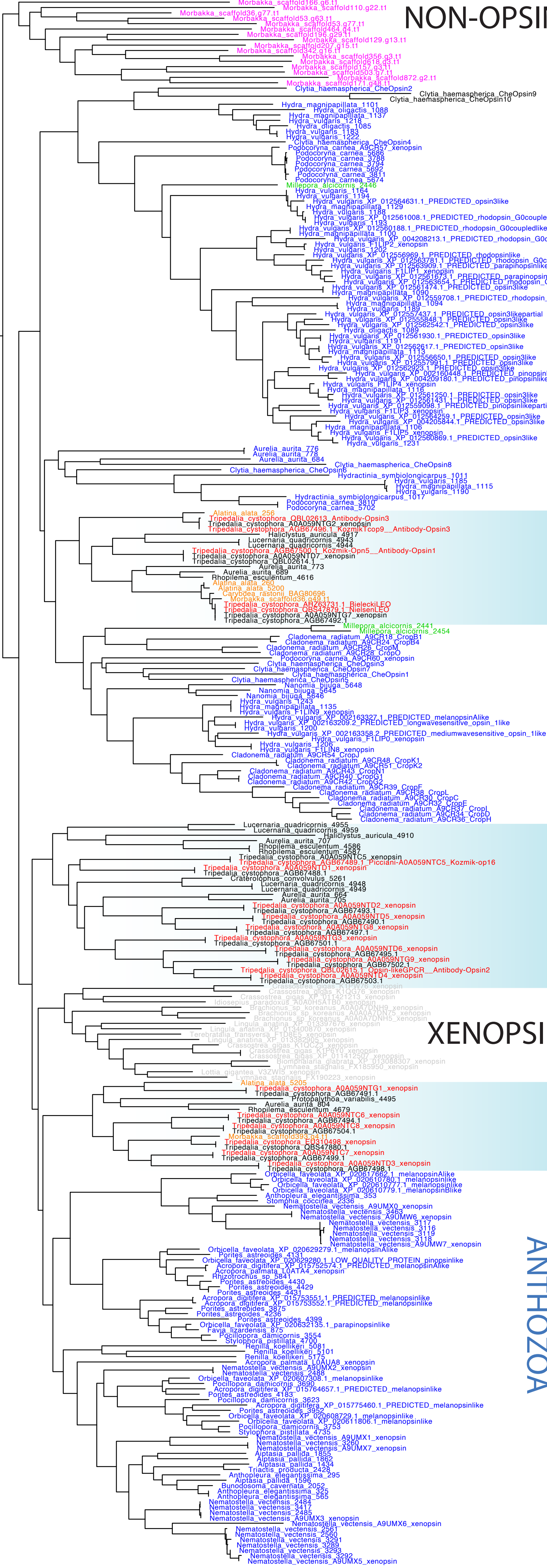

Supplement: Supplementary Figure 2 — Full phylogenetic tree for our xenopsin + cnidops dataset. See Supplementary Figure 1 and main text for methodological details. The scale bar indicates amino acid substitutions per site. [file Data_Sheet_2.PDF]
