# Supplementary material for: Expression of Opsins of the Box Jellyfish Tripedalia cystophora Reveals the First Photopigment in Cnidarian Ocelli and Supports the Presence of Photoisomerases
Source: Front Neuroanat. 2022 Aug 5;16:916510. doi: 10.3389/fnana.2022.916510 (PMC9389615; doi:10.3389/fnana.2022.916510)
Supplement: Supplementary file 3 [file Data_Sheet_3.PDF]

Table S1. Tripedalia opsin gene names.

Gene Clade	GenBank	UniProt	Synonymous Names	Protein	RNA Expression
2**	QBL02613.1; AGB67496.1	A0A481ZM56; A0A059NTG2	GCO*, Ab-Opsin3*; Tcop9**	Cnidocytes, gonads*	Rhopalia**
2**		A0A059NTD7	SEO*, Ab-Opsin1*; Tcop5 **	Slit eyes*	Rhopalia**
2**	QBS47879.1; AHZ63731.1; AGB67492.1	A0A059NTG7	LEO#; Tcop13**	Lens Eye**	Lens Eye#, Rhopalia**
2**	AGB67492.1	A0A059NTG7	Tcop13**		Rhopalia**
1b	QBL02615.1	A0A481ZLS6	GEO*, Ab-Opsin2*	All eyes*	
1b**	AGB67503.1	A0A059NTD4	Tcop2**		Rhopalia,gonads**
1b**	AGB67502.1	A0A059NTG9	Tcop3**		Rhopalia**
1b**	AGB67495.1	A0A059NTD6	Tcop10**		Gonads**
1b**	AGB67501.1	A0A059NTG3	Tcop4**		Rhopalia**
1b**	AGB67497.1	A0A059NTG8	Tcop8**		Manubrium
1b**	AGB67493.1	A0A059NTD2	Tcop12**		Tentacles,Outer umbrella**
1b**	AGB67490.1	A0A059NTD5	Tcop15**		Tentacles, gonads**
1b**	AGB67489.1	A0A059NTC5	Tcop16**		Rhopalia**
1b**	AGB67488.1	A0A059NTD1	Tcop17**		Rhopalia,gonads**
1a**	AGB67504.1	A0A059NTC8	Tcop1**		Rhopalia**
1a**	AGB67499.1	A0A059NTC7	Tcop6**		Rhopalia**
1a**	AGB67498.1	A0A059NTD3	Tcop7**		Rhopalia**
1a**	AGB67494.1	A0A059NTC6	Tcop11**		Rhopalia**
1a**	AGB67491.1	A0A059NTG1	Tcop14**		Tentacles**
1a**	QBS47880.1	A0A4D5XWB3	NEO#; Tcop18**		Neuropil#, Rhopalia**

* This study
** Liegertová et al., 2015
*** Nielsen et al., 2019
\# Bielecki et al., 2014
\## Picciani et al., 2018
